# Selection, Expansion, and Unique Pretreatment of Allogeneic Human Natural Killer Cells with Anti-CD38 Monoclonal Antibody for Efficient Multiple Myeloma Treatment

**DOI:** 10.3390/cells10050967

**Published:** 2021-04-21

**Authors:** Benjamin Motais, Sandra Charvátová, Zuzana Walek, Matouš Hrdinka, Ryszard Smolarczyk, Tomasz Cichoń, Justyna Czapla, Sebastian Giebel, Michal Šimíček, Tomáš Jelínek, Tereza Ševčíková, Jiří Sobotka, Zdeněk Kořístek, Roman Hájek, Juli R. Bagó

**Affiliations:** 1 Faculty of Medicine, University of Ostrava, 703 00 Ostrava, Czech Republic; benjamin.motais@fno.cz (B.M.); sandra.charvatova@fno.cz (S.C.); zuzana.walek@fno.cz (Z.W.); matous.hrdinka@fno.cz (M.H.); michal.simicek@fno.cz (M.Š.); tomas.jelinek@fno.cz (T.J.); tereza.sevcikova@fno.cz (T.Š.); zdenek.koristek@fno.cz (Z.K.); roman.hajek@fno.cz (R.H.); 2 Faculty of Science, University of Ostrava, 701 00 Ostrava, Czech Republic; 3 PrimeCell Advanced Therapy, Inc., 708 52 Ostrava, Czech Republic; 4 Department of Haematooncology, University Hospital Ostrava, 708 00 Ostrava, Czech Republic; 5 Center for Translational Research and Molecular Biology of Cancer, Maria Sklodowska-Curie National Research Institute of Oncology, Gliwice Branch, 44 102 Gliwice, Poland; Ryszard.Smolarczyk@io.gliwice.pl (R.S.); tcichon@io.gliwice.pl (T.C.); justyna.czapla@io.gliwice.pl (J.C.); 6 Department of Bone Marrow Transplantation and Onco-Hematology, Maria Sklodowska-Curie National, Research Institute of Oncology, Gliwice Branch, 44 102 Gliwice, Poland; Sebastian.Giebel@io.gliwice.pl; 7 Spadia LAB, a.s., Frenštát pod Radhoštěm, 700 30 Ostrava, Czech Republic; jiri.sobotka@spadia.cz

**Keywords:** cancer, natural killers, cellular therapy, monoclonal antibodies, multiple myeloma, combination therapy

## Abstract

Cellular immunotherapy is becoming a new pillar in cancer treatment after recent striking results in different clinical trials with chimeric antigen receptor T cells. However, this innovative therapy is not exempt from challenges such as off-tumor toxicity, tumor recurrence in heterogeneous tumors, and affordability. To surpass these limitations, we exploit the unique anti-tumor characteristics of natural killer (NK) cells. In this study, we aimed to obtain a clinically relevant number of allogeneic NK cells derived from peripheral blood (median of 14,050 million cells from a single donor) to target a broad spectrum of solid and liquid tumor types. To boost their anti-tumor activity, we combined allogeneic NK cells with the approved anti-cluster of differentiation 38 (CD-38) monoclonal antibody Daratumumab to obtain a synergistic therapeutic effect against incurable multiple myeloma. The combination therapy was refined with CD16 polymorphism donor selection and uncomplicated novel in vitro pretreatment to avoid undesired fratricide, increasing the in vitro therapeutic effect against the CD-38 positive multiple myeloma cell line by more than 20%. Time-lapse imaging of mice with established human multiple myeloma xenografts revealed that combination therapy of selected and pretreated NK cells with Daratumumab presented tumor volumes 43-fold smaller than control ones. Combination therapy with an allogeneic source of fully functional NK cells could be beneficial in future clinical settings to circumvent monoclonal antibodies’ low therapeutic efficiency due to NK cell dysfunctionality in MM patients.

## 1. Introduction

The introduction of novel therapeutic approaches to treat cancer based on immune cells such as chimeric antigen receptor (CAR)-T cells has revolutionized the battle against cancer. Striking efficacy of CAR-T cells in clinical trials to treat different hematological malignancies [1,2,3] is consolidating cancer cell-based immunotherapy as a new pillar in cancer treatment. This initial success paved the way for the exploration of new cell-based immunotherapies to circumvent limitations inherent to CAR-T cells, such as on-target off-tumor toxicity [4], tumor escape by loss of CAR-targeted antigen in tumor cells [5], and the requirement for the autologous T cells to avoid life-threatening complications such as graft-versus-host-disease (GvHD) [6]. These limitations have fostered the exploration of other immune cells as therapeutic vehicles for cancer treatment, such as NK cells [7], macrophages [8], or dendritic cells [9]. Amongst the different candidates, NK cells present exceptional anti-tumor activities and the capacity to exert immune response against tumor cells through mechanisms other than those imposed by a specific CAR [10], potentially reducing the risk of tumor escape by loss of the CAR-targeted antigen. The NK cells express inhibitory and activating receptors that control the cytotoxic response after engagement with specific antigens present in tumor cells [11,12]. NK cells’ antitumor activity can also be triggered by the engagement of growth factors produced by tumor cells with specific activator receptors in NK cells [13]. Importantly, NK cells have low on-target off-tumor toxicity and lack the potential to produce GvHD in allogeneic sources [14], providing a readily available “off-the-shelf” product.

These unique anti-tumor features of NK cells make them an excellent candidate for the next generation of cancer immunotherapy. In 2021, the ClinicalTrials.gov database registered more than 20 clinical trials utilizing NK cells to treat MM. They are in phase I and II and can be divided into two main groups depending on NK cell source, autologous or allogeneic. Regarding allogeneic sources, the NK cells in the actual clinical trials are derived from the NK92 cell line [15], cord blood [16], and peripheral blood [17]. To date, no severe adverse events have been reported in the different studies. Although results are still too preliminary to conclude efficacy, a recently disclosed clinical trial in phase II using allogeneic cord-blood derived NK cells with lenalidomide and autologous stem cell transplantation reported 83% complete response or very good partial response in 33 patients [18].

Because cancer cell-based therapy with NK cells is still in its infancy, further characterization of the cell source, expansion capacity, safety, and efficacy will contribute to future clinical success in patients’ treatment.

In this manuscript, we aimed to produce a clinically relevant NK cells dose with broad efficacy against different cancer types. To this end, we compared the reproducibility in the isolation and expansion of NK cells derived from peripheral blood (PBNK cells) and assessed their therapeutic potential in vitro by analyzing their anti-tumor activity and migration capacity towards different tumor cell lines. To boost the anti-tumor activity of PBNK cells against multiple myeloma (MM) cells without arduous genetic manipulations, we decided to combine PBNK cells with the clinically approved monoclonal antibody Daratumumab [19]. The isolated and expanded PBNK cells express the FcγRIII receptor (CD16), which is capable of binding human immunoglobulin G (IgG) Fc in monoclonal antibodies and triggering the antibody-dependent cell-mediated cytotoxicity (ADCC) against tumor cells [20]. The combinatorial therapy with expanded PBNK cells and Daratumumab produces a synergistic effect, significantly increasing the cytotoxicity against multiple MM cancer cells. Furthermore, combinatorial therapy was enhanced after selecting specific PBNK cell donors with a particular CD16 allotype [21] and a novel and straightforward culture method to avoid fratricide.

Our current study demonstrates the feasibility and reproducibility of obtaining clinically relevant concentrations of medical-grade PBNK cells. The PBNK cells generated by our protocol target a broad spectrum of tumor cell types, and their therapeutic efficacy can be boosted with clinically approved monoclonal antibodies (mAbs). The combination therapy with fully functional allogeneic PBNK cells could represent a solution to the low therapeutic efficiency of mAbs in a single-agent treatment due to inherent NK cell dysfunctionality in patients with MM [22].

## 2. Materials and Methods

### 2.1. Ethics Statement

Peripheral blood mononuclear cells (PBMC) were obtained from healthy donors at the Blood Center of the University Hospital of Ostrava with the ethics committee’s approval. PBMC and bone marrow aspirates were obtained from patients with newly diagnosed MM after signing informed consent approved by the institutional ethical committee.

### 2.2. Isolation of NK Cells from Peripheral Blood and Abnormal Plasmatic Cells from Bone Marrow Aspirates

PBMC from healthy donors and patients were isolated by density gradient centrifugation (Ficoll Paque PLUS, density 1.077 g/mL, GE Healthcare, Chicago, IL, USA). Then, we negatively enriched NK cells with an NK Cell Isolation Kit (Miltenyi, Bergisch Gladbach, Germany) using the AutoMACS Pro (Magnetic-Activated Cell Sorting, Miltenyi, Bergisch Gladbach, Germany, version 2.3.0.2), following the manufacturer’s protocol.

Bone marrow mononuclear cells (BMMC) were isolated from bone marrow aspirates by density centrifugation (Ficoll Paque PLUS, density 1.077 g/mL, GE Healthcare, Chicago, IL, USA), and abnormal plasmatic cell (aPC) enrichment was performed with CD138 MicroBeads (Miltenyi, Bergisch Gladbach, Germany) using the AutoMACS Pro, following the manufacturer’s protocol. The phenotype of separated aPC was verified by FACSAria III (BD Biosciences-US, San Jose, CA, USA, version 8.0.1) using the following fluorescent antibodies: CD19-PECy7 (Beckman Coulter, Brea, CA, USA), CD56-PE (Agilent Dako, Santa Clara, CA, USA), CD38-FITC (Cytognos, Salamanca, Spain), CD138-APC (Sony, San Jose, CA, USA), and CD45-PB (Agilent Dako, Santa Clara, CA, USA).

### 2.3. Cell Culture, Pretreatment with Daratumumab, and Induction of Interferon Gamma (IFN-γ) Expression by Increasing Concentrations of Platelet-Derived Growth Factor (PDGF-DD)

PBNK cells were cultured in non-adherent flasks with complete NK MACS medium (Miltenyi, Bergisch Gladbach, Germany) supplemented with 5% of human AB serum (Sigma-Aldrich, Munich, Germany), 500 U/mL of interleukin-2 (IL-2; Peprotech, Cranbury, NJ, USA), 100 ng/mL of interleukin-15 (IL-15; Peprotech, Cranbury, NJ, USA), and 0.5% of penicillin-streptomycin (Sigma-Aldrich, Munich, Germany). The medium was changed twice a week, and cells were routinely counted using the Luna-Stem Dual Fluorescence Cell Counter (Logos Biosystems, Anyang, Korea, version 1.4.0) to maintain a concentration of 1 × 10^6^ to 2 × 10^6^ cells/mL. To avoid fratricide, PBNK cells were cultivated for 48 h with Daratumumab at a final concentration of 10 µg/mL.

The induction of IFN-γ expression in PBNK cells with PDGF-DD was studied after 16 h of incubation with increasing concentrations of recombinant human PDGF-DD (R&D Systems, Minneapolis, MN, USA) (0 ng/mL, 5 ng/mL, 10 ng/mL, and 25 ng/mL). To further support specific PDGF-DD/NKp44 interaction in the IFN-γ induction, PBNK cells were incubated for 16 h with 5 ng/mL PDGF-DD in the presence of 5 μg/mL IgG1 (Sony, San Jose, CA, USA) or blocking anti-NKp44 mAb (Miltenyi, Bergisch Gladbach, Germany). The amount of IFN-γ under the different conditions was quantified with the human IFN-γ ELISA Kit (Invitrogen, Waltham, MA, USA) following the manufacturer’s protocol.

Our panel of cancer cells consisted of human MM cell lines RPMI 8226 (ATCC CCL-155) and U266 (ATCC TIB-196), the human lung carcinoma cell line A549 (a gift from Dr. Peter Dráber, BIOCEV, Prague, Czech Republic), human triple-negative breast cancer MDA-MB-231 (ATCC HTB-26), human myeloid leukemia cell line K562, and human osteosarcoma cell line U-2 OS (provided by Prof. Václav Hořejší, Institute of Molecular Genetics, Prague, Czech Republic). All cell lines, except for U-2 OS, were cultured in the RPMI 1640 medium (Sigma-Aldrich, Munich, Germany) supplemented with 10% of FBS (Sigma-Aldrich, Munich, Germany), 1% of Ultraglutamine-1 (Lonza, Basel, Switzerland), and 1% of penicillin-streptomycin. U-2 OS cells were grown in high-glucose DMEM (Sigma-Aldrich, Munich, Germany) with 10% of FBS and 1% of penicillin-streptomycin.

### 2.4. Phenotypic and Functional Analyses of Expanded PBNK Cells

To evaluate the expression of NK cell phenotype markers, we performed flow cytometry analysis (BD FACSAria^TM^ III Cell Sorter (BD Biosciences-US, San Jose, CA, USA, version 2.3.0.2)) of expanded PBNK cells with a panel of antibodies as previously described [23]. The panel consisted of CD56-APC-Cy7-Vio770 (Miltenyi, Bergisch Gladbach, Germany), CD16-APC (Miltenyi, Bergisch Gladbach, Germany), NKG2A-PE-Vio770 (Miltenyi, Bergisch Gladbach, Germany), NKG2C-PE (Miltenyi, Bergisch Gladbach, Germany), NKG2D-PerCP-Cy5.5 (Biolegend, San Diego, CA, USA), KIR2D-FITC (Miltenyi, Bergisch Gladbach, Germany), and SYTOX^®^ Blue Dead Cell Stain (Invitrogen, Waltham, MA, USA) to evaluate cell viability. Non-NK cell lineage markers were also analyzed with the following antibodies: CD3-VioBlue (Miltenyi, Bergisch Gladbach, Germany), TCRg/d-VioBlue (Miltenyi, Bergisch Gladbach, Germany), CD14-VioBlue (Miltenyi, Bergisch Gladbach, Germany), and CD19-VioBlue (Miltenyi, Bergisch Gladbach, Germany). The analysis of polymorphism on FcγRIIIA-158 was performed with two different mAbs, as previously described [24]. One mAb that recognizes both FcγRIIIA-158 polymorphisms (V/F) (3G8 clone, FITC-conjugated (Southern Biotech, Birmingham, AL, USA)), and other mAb that detect only one FcγRIIIA-158 polymorphism (V) (clone MEM-154 conjugated with APC (Novus Biotech, Centennial, CO, USA)). MEM-154/3G8 median fluorescence intensity (MFI) ratios were calculated to characterize the proper allotype in PBNK cells, resulting in F/F in ratios < 0.04, V/F in ratios ranging from 0.15 to 0.48, and V/V in ratios higher than 0.64. The expression of three cytotoxic markers (IL-2 receptor: CD25-VioBright-FITC (Miltenyi, Bergisch Gladbach, Germany), lysosome-associated membrane glycoprotein: CD107a-PE (Miltenyi, Bergisch Gladbach, Germany), and natural cytotoxicity-triggering receptor: NKp44-PE-Vio770 (Miltenyi, Bergisch Gladbach, Germany)) were analyzed by FACS after cultivation of 1.5 × 10^5^ PBNK cells with 1.5 × 10^5^ cells (RPMI 8226, K-562, A549, U-2 OS, or MDA-MB-231) for 24 h.

### 2.5. Lentiviral Vectors

The lentiviral construct for the constitutive expression of the red fluorescence (mCherry; mCh) and bioluminescence (firefly luciferase; FLuc) reporters was generated by amplifying the cDNA encoding both reporters from Addgene Plasmid #44965 and cloned in a lentivirus backbone (#12262, Addgene, Watertown, MA, USA) using standard cloning procedures.

The lentivirus construct was packaged as a lentivirus vector in human embryonic kidney 293FT cells (HEK 293FT, a kind gift from Prof. Vaclav Hořejší, Institute of Molecular Genetics, Prague, Czech Republic). 293FT cells were seeded at 3–4 × 10^6^ cells per 10 cm dish in complete DMEM without antibiotics. On the next day, the cells were transfected with the dual reporter plasmid, the psPAX2 packaging plasmid (#12260, Addgene, Watertown, MA, USA), and the pMD2.G plasmid encoding the VSV-G envelope (#12259, Addgene, Watertown, MA, USA) using the jetPRIME transfection system (Polyplus, Illkirch-Graffenstaden, France). The medium was replaced five hours after transfection, and the viral supernatant was collected 64 h later. After being filtered with a 0.45 µm filter, the virus was concentrated from supernatants with Amicon Ultra-15 filter tubes (Millipore, Burlington, MA, USA). The different tumor cell lines were infected with the concentrated virus at a varying multiplicity of infection in culture media containing 8 µg/mL of Polybrene (Sigma-Aldrich, Munich, Germany).

### 2.6. Cytotoxic Activity of PBNK Cells and Daratumumab-Mediated Cytotoxicity

Cytotoxic assays of expanded PBNK cells against different tumor cells lines were performed in quadruplicate in flat white 96-well plates with a clear bottom (Thermo Scientific, Waltham, MA, USA). In each well, 2 × 10^4^ tumor cells (target cells) labeled with luciferase expression were mixed with 2 × 10^4^ PBNK cells (effector cells) in a final volume of 120 µL RPMI 1640 complete medium (1:1 effector-target ratio). To assess the Daratumumab-mediated cytotoxicity, Daratumumab (Janssen, Beerse, Belgium) was added to the wells at a final concentration of 10 µg/mL. In all cases, the number of target cells was assessed 24 h later by adding d-luciferin potassium salt (Goldbio, St. Louis, MO, USA) to a final concentration of 0.5 mg/mL in the wells and immediately measuring the bioluminescence with the Infinite F Plex microplate reader (Tecan, Männedorf, Switzerland, version 3.9.0.1). Bioluminescence peak values were measured in each well, and the specific lysis percentage was calculated using the following equation, as previously described [25]:

(1) $$\%\;specific\;lysis=\frac{mean\;BLI\;control-BLI\;replicate\;sample}{mean\;BLI\;control}*100$$

Cytotoxic assays of autologous and allogeneic PBNK cells against aPCs from patients with MM were performed in triplicate in 24 well plates for four hours. The aPCs (2 × 10^5^ cells) were mixed with 10 × 10^5^ autologous or allogenic PBNK cells (5:1 effector-target ratio) in a final volume of 1 mL RPMI 1640 complete medium. Daratumumab (Janssen, Beerse, Belgium) was added to the wells at a final concentration of 10 µg/mL. To calculate the specific lysis after coculture, the absolute number of aPCs under the different conditions was calculated by FACSAria III (BD Biosciences-US, San Jose, CA, USA, version 8.0.1) using specific fluorescent antibodies (CD19-PECy7 (Beckman Coulter, Brea, CA, USA), CD56-PE (Agilent Dako, Santa Clara, CA, USA), CD38-FITC (Cytognos, Salamanca, Spain), and CD45-PB (Agilent Dako, Santa Clara, CA, USA)), and dead cells were discriminated by 7-AAD Viability Staining (Invitrogen, Waltham, MA, USA). To assure the accuracy of the absolute count results, flow cytometry analysis was performed with Perfect Count Microspheres (Cytognos, Salamanca, Spain). The specific lysis percentage was calculated using the following equation:

(2) $$\%\;specific\;lysis=\frac{Mean\;number\;of\;live\frac{aPC}{µL}\;in\;control-Number\;of\;live\frac{aPC}{µL}\;\mathrm{in}\;\mathrm{coculture}}{Mean\;number\;of\;live\frac{aPC}{µL}\;in\;control}*100$$

### 2.7. Quantification of IFN- γ and PDGF-DD 

The expression of IFN-γ in PBNK cells after coculture with different cancer cell lines or after incubated with increasing PDGF-DD concentrations was quantified with the human IFN-γ ELISA Kit (Invitrogen, Waltham, MA, USA) following the manufacturer’s protocol. The expression of PDGF-DD in the tumor cell lines was quantified with the human PDGF-DD DuoSet ELISA kit (R&D Systems, Minneapolis, MN, USA) following the manufacturer’s protocol.

### 2.8. Migration Assay

The migration capacity of expanded PBNK cells was evaluated in chemotaxis transwell assays with cancer cell line serum-free conditioned media from RPMI 8226, K562, A549, MDA-MB-231, and U-2 OS (48 h). Briefly, transwell permeable supports (Costar) with 5 µm pores and a 6.5 mm diameter were placed on top of a 24-well plate containing 500 µL of conditioned medium from each cancer cell line. Expanded and serum-starved (24 h) PBNK cells were seeded in 200 µL of medium without serum in the upper chamber, at a density of 1.5 × 10^5^ cells/well. Twenty-four hours later, PBNK cells that migrated through the pores were collected from the bottom chamber and counted using the Luna-Stem Dual Fluorescence Cell Counter (Logos Biosystems, Anyang, Korea, version 1.4.0).

### 2.9. In Vivo Experiments

All animal-related procedures were performed with the approval of the Animal Care Committee at the Maria Sklodowska-Curie National Research Institute of Oncology in Gliwice, Poland.

For the in vivo study, adult 6–8-week-old female severe combined immunodeficient mice (SCID) (lack of T and B lymphocytes and impaired NK cells) were purchased from Charles River Laboratories (Wilmington, MA, USA). Following 14 days acclimatization, subcutaneous injection of 100 µL of PBS containing 1 × 10^7^ RPMI 8226 cells (mCh-FLuc) mixed with 100 µL of Matrigel (Corning, Corning, NY, USA) was performed in the right rear flank of 16 SCID mice. To determine the in vivo therapeutic efficacy of expanded PBNK cells in combination with mAbs, the mice were divided into three different treatment groups: control group without treatment, single-agent treatment with PBNK cells, and combinatorial treatment group with Daratumumab and PBNK cells. On days 7 and 14 after tumor implantation, the mice received the corresponding regimen treatment. The single-agent treatment group was treated with a tail vein injection of 1 × 10^7^ PBNK cells in 200 µL of PBS, using a 28-gauge needle syringe. The combinatorial treatment group received a tail vein injection of 200 µL of PBS containing 1 × 10^7^ PBNK cells with 8 mg/kg Daratumumab. The control group received a tail vein injection of 200 µL PBS. On day 1 and every seven days after tumor implantation, bioluminescence imaging was performed in all groups to monitor tumor progression. To this end, the intraperitoneal injection of D-luciferin (1.5 mg/mouse) was given to mice, and photon emission was registered five minutes later using an IVIS Lumina XR In Vivo Imaging System (PerkinElmer, Waltham, MA, USA). Exposure time was adjusted depending on the obtained signal in each case. The images were processed and photon emission quantified by measuring average radiance (p/s/cm^2^/cr) using LivingImage software (PerkinElmer, Waltham, MA, USA, version 4.1.0.11858). Thereafter, mice were returned to the cages and monitored for a few minutes. The mice were monitored on a daily basis for survival analysis in the different groups. Mice with considerable weight loss (>20% of their initial body weight) or signs of pain (rough hair coat or hunched posture) were sacrificed.

### 2.10. Statistical Analysis

Our data were analyzed with GraphPad Prism 5.00 for Windows (San Diego, CA, USA). Student’s two-tailed t-test and one-way ANOVA were used when appropriate to determine statistical significance. P values were considered statistically significant when *p* < 0.05.

## 3. Results

### 3.1. Isolation and Phenotypic Characterization of Expanded PBNK Cells

**Figure 1:**
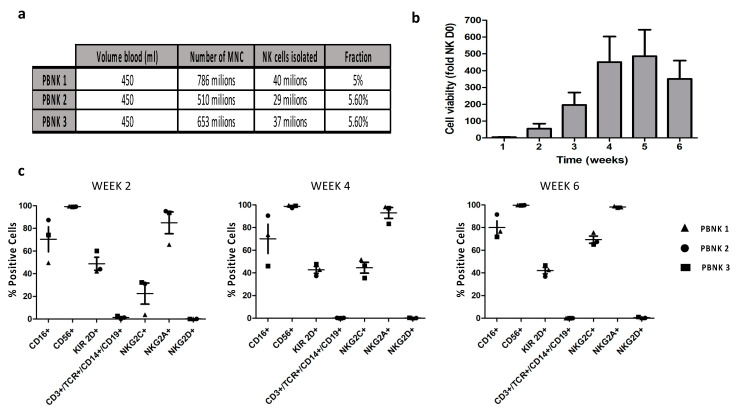
Isolation of NK cells, expansion to clinically relevant PBNK cell concentrations, and phenotype analysis. (**a**) A representative table with the yields of PBNK cells from three different donors using a negative selection approach. (**b**) Summary graph showing the median growth fold of the three PBNK cell donors isolated and expanded in the feeder-free NK MACS medium. (**c**) Representative graphics of PBNK cell purity obtained by FACS at the indicated number of days post-expansion. Data in (**b**,**c**) are means ± SEM of the three expanded PBNK cell donors.

PBNK cells were isolated from three healthy donors using negative selection with the untouched NK cell isolation kit (Miltenyi, Bergisch Gladbach, Germany). In all cases, we obtained a similar number of PBNK cells, accounting for ~5% of mononuclear cells (Figure 1a). Expansion of the NK cells was performed without feeder cells in the NK MACS Medium (Miltenyi, Bergisch Gladbach, Germany) supplemented with IL-2 and IL-15. Cell proliferation and viability were tracked for six weeks, reaching the maximum cell number in the fifth week, with an average fold expansion of 486 ± 157.8 (Figure 1b) and a median of 14,050 ± 302 million PBNK cells from a single donor. The resulting doubling times were 101 h, 47 h, and 40 h for PBNK cells from donors 1, 2, and 3, respectively. Next, along the time of expansion, we conducted the phenotypic characterization of the three expanded PBNK cell donors by flow cytometry using antibody panels to cover non-NK lineage and NK cell lineage markers. During the six weeks of expansion, we observed a lack of expression of non-NK lineage markers and positive expression of the distinct markers related to NK cell lineage in all three expanded PBNK cell donors (Figure 1c). Interestingly, most of the different markers remained stable during the six weeks of the expansion period, except for the NK phenotype marker NKG2C, which significantly and progressively increased over time in all three donors. The NKG2C receptor is an activating receptor in NK cells and, together with the inhibitory receptor NKG2A, belongs to the family of the C-type lectin receptors [26]. The ligand for these receptors is the non-classical HLA-E molecule, frequently overexpressed in different types of tumor cells [27,28].

Collectively, our results indicate the feasibility and reproducibility in the isolation and expansion of PBNK cells, achieving highly purified and clinically relevant numbers of NK cells after five weeks of expansion, applying a straightforward feeder-free method of expansion.

### 3.2. Functional Characterization of Expanded PBNK Cells

**Figure 2:**
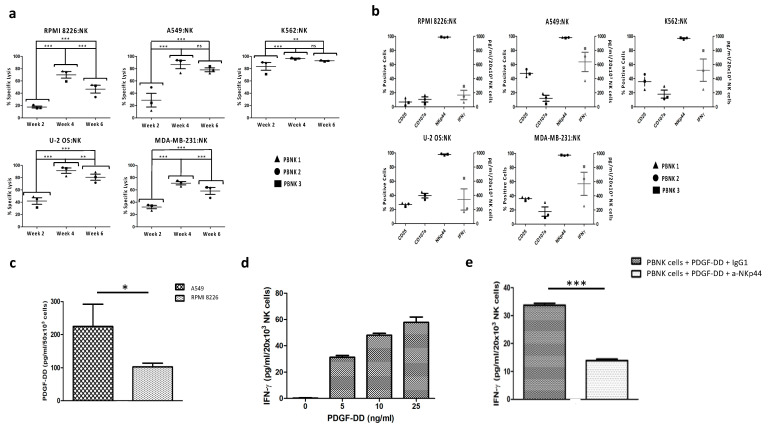
Cytotoxicity of expanded PBNK cells and expression of cytotoxicity-associated molecules. (**a**) The cytotoxicity of three expanded PBNK cell donors towards RPMI 8226, A549, K562, U-2 OS, and MDA-MB-231 measured using the bioluminescence imaging assay at a 1:1 effector-to-target ratio for 24 h. (**b**) Expression of cytotoxicity-associated molecules (CD25, CD107a, NKp44, and IFN- **γ**) in PBNK cells in the fourth week of expansion after coculture with the different cancer cell lines (RPMI 8226, A549, K562, U-2 OS, and MDA-MB-231) at a 1:1 effector-to-target ratio for 24 h. (**c**) Expression of PDGF-DD in A549 and RPMI 8226. (**d**) Histogram with IFN-**γ** expression by PBNK cells after stimulation with different concentrations of PDGF-DD. (**e**) Graphic with IFN-**γ** expression by PBNK cells after stimulation with 5 ng/mL of PDGF-DD and IgG1, and after stimulation with 5 ng/mL of PDGF-DD and the anti-NKp44 mAb. Data in (**a**,**b**) are means ± SEM of the three expanded PBNK cell donors. Data in (**c**–**e**) are presented as means ± SD from three technical replicates of a PBNK cell pool. ns, not significant, *, *p* < 0.05, **, *p* < 0.01, ***, *p* < 0.001, one-way ANOVA, repeated measures test and Student’s *t*-test.

Next, we evaluated the ability of the PBNK cells isolated and expanded from three different donors to mediate activity against different tumor cells. For this purpose, we performed a bioluminescence-based cytotoxicity assay [25] based on the co-culture of the expanded PBNK cells with several human tumor target cell lines stably transduced to express luciferase. The specific lysis of target cell lines by PBNK cells was measured in weeks two, four, and six of expansion. As shown in Figure 2a, we observed cytotoxicity against the five targeted tumor cell lines for the different expanded PBNK cell donors, achieving maximum cytotoxicity in the fourth week of expansion. In week six, the specific lysis tended to decrease in all three expanded PBNK cell donors, most likely and concordantly with the literature, due to prolonged exposure to IL-15 that entails NK cell exhaustion [29]. As expected, the most potent cytotoxic activity was observed against the cancer cell line K562 cells, followed by the U-2 OS and A549 cell lines. The higher reactivity of the expanded PBNK cells against the erythroleukemic cell line K562 is due to the lack of expression on those tumor cells of MHC class-I inhibitory ligands [30].

In accordance with these results, the cytotoxic activity of PBNK cells in the fourth week of expansion was linked to the expression of molecules involved in NK cell-mediated cytotoxicity, such as IFN-γ, degranulation marker differentiation CD107A, and the NK activation receptors CD25 and NKp44 (Figure 2b). Among our three PBNK cell donors, the PBNK 1 cells expressed a significantly lower amount of IFN-γ against the different target tumor cell lines as compared to the other two PBNK cell donors. This lower expression was compensated by a higher expression of the degranulation marker CD107A, resulting in similar cytotoxicity in all three expanded PBNK cell donors.

A recent study pointed out the activation of the NKp44 receptor in NK cells after engagement with PDGF-DD expressed by tumor cells [13]. The PDGF-DD-NKp44 interaction triggers IFN-γ and tumor necrosis factor-alpha expression in NK cells, inducing tumor cell growth arrest. Because expanded PBNK cells have a high expression of NKp44, we determine whether this mechanism of IFN-γ induction was present in expanded PBNK cells. To this end, we first analyzed the PDGF-DD production by tumor cell lines RPMI 8826 and A549, with a lower and a higher expression of IFN-γ after coculture, respectively. As shown in Figure 2c, we observed a significantly higher expression of PDGF-DD in A549 than in RPMI 8826, which correlates with the differences in IFN-γ expression observed in cocultures with PBNK cells.

We next demonstrated the effect of the PDGF-DD-NKp44 interaction on the PBNK cell expression of IFN-γ. PBNK cells were incubated with increasing PDGF-DD concentrations to observe the dose-dependent expression of IFN-γ (Figure 2d). To further support the link between IFN-γ expression and the PDGF-DD/NKp44 interaction, we confirmed the significant block of IFN-γ expression in PBNK cells incubated with PDGF-DD and the anti-NKp44 antibody (Figure 2e)

Collectively, the data obtained indicate a broad cytotoxic activity of the expanded PBNK cells towards different tumor cell lines. This cytotoxic activity was similar in PBNK cells from three different donors, reaching the maximum in the fourth week of expansion. We proved that, at least in part, the induction of IFN-γ expression was mediated by the engagement of the tumor cell growth factor PDGF-DD with the NKp44 receptor in PBNK cells.

### 3.3. Tumor-Homing of Expanded PBNK Cells

NK cells have an inherent capacity to migrate and infiltrate different cancers. The homing of expanded PBNKs to tumor target cells is essential for an efficient anti-tumor response, and the therapeutic outcomes have been directly linked to the ability of these NKs to home the tumor [31,32,33]. The migration of the NK cells to tumors is known to be induced by chemotaxis triggered by the binding of NK cell receptors to chemokines secreted by tumor cells and the tumor microenvironment [34].

**Figure 3:**
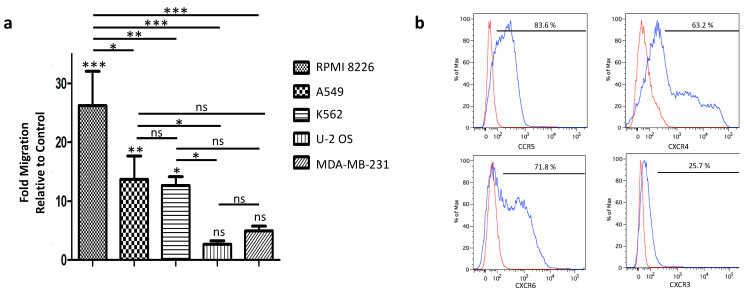
Migration capacity of expanded PBNK cells towards different cancer cell lines and their chemokine receptor analysis. (**a**) Summary graph of the transwell migration assay of expanded PBNK cells towards different cancer cell conditioned media (RPMI 8226, A549, K562, U-2 OS, and MDA-MB-23). (**b**) FACS analysis of PBNK cells for chemokine receptors CXCR3, CXCR4, CCR5, and CXCR6 (blue) and the isotype-matched controls (red). Data in (**a**) are presented as means ± SEM of three independent experiments performed in triplicate. ns, not significant, *, *p* < 0.05, **, *p* < 0.01, ***, *p* < 0.001, one-way ANOVA and repeated measures test.

We performed a transwell migration assay in different tumor-conditioned media to assess the migration capacity of PBNK cells (Figure 3a). In all five conditioned media tested, we observed a significant increase in the PBNK cells’ migration capacity. This increase in migration was exceptionally high in conditioned medium from the MM cell line RPMI 8226. Next, we analyzed the expression levels of chemokine receptors in PBNK cells and corresponding chemotaxis ligands in tumor cells. In this regard, as shown in Figure 3b, we found that the PBNK cells expressed the well-known chemotaxis receptors CXCR3, CXCR4, CCR5, and CXCR6, which are activated by chemokines secreted by the cancer cell lines [35]. Therefore, the observed higher migration of PBNK cells towards RPMI 8226 cells can be explained by the secretion of CXCL9, CXCL10, and CXCL12 [36], the ligands for the chemotaxis receptors CXCR3 and CXCR4 expressed in PBNK cells. Thus, the results presented here demonstrate the migration capacity of expanded PBNK cells towards different types of tumor cells, with a significant higher tumor-tropism towards the MM cell line RPMI 8226.

### 3.4. Combinatorial Treatment of Expanded PBNK Cells with Monoclonal Antibodies

The use of mAbs for cancer treatment has become one of the most successful approaches in hematological and solid malignancies [37]. The mechanisms of action are diverse and, in some cases, include antibody-dependent cell-mediated cytotoxicity (ADCC) [38], opening the possibility of combining the mAbs with the expanded PBNK cell therapy as an efficacy’s multiplier. In NK cells, ADCC is regulated by the FcγRIII (CD16) receptor encoded by the *FCGR3A* gene. A single nucleotide polymorphism on this gene results in two allotypes of the receptor, one with valine (V) and another with phenylalanine (F) at the amino acid 158. NK cells with at least one V in the two alleles have a stronger affinity to IgG_1_ and, consequently, a higher ADCC [39,40]. In an attempt to select the PBNK cells with a better potential to cooperate with the mAbs, we performed a flow cytometry assay in expanded PBNK cells with two specific antibodies [24]; one that is able to recognize the FcγRIIIA-158 V polymorphism (MEM-154) and another that detects both FcγRIIIA-158 V/F polymorphisms (3G8). The ratio resulting from the median fluorescence intensity (MFI) of both antibodies (154/3G8) can faithfully predict the *FCGR3A* genotype (F/F, F/V, or V/V).

**Figure 4:**
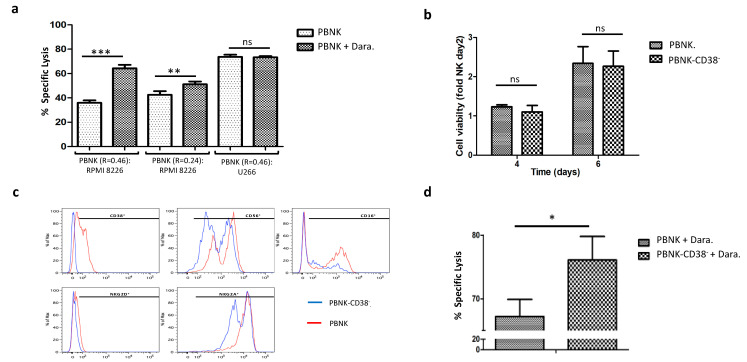
Selection and pretreatment of expanded PBNK cells with Daratumumab to increase synergy in combinatorial therapy with mAbs. (**a**) Graph with the specific lysis results in a combinatorial therapy with PBNK cells (R = 0.46 and R = 0.24) and Daratumumab against MM cell lines RPMI 8226 and U266. Measured using the bioluminescence imaging assay at a 1:1 effector-to-target ratio for 24 h. (**b**) Summary graph with the viability over time of PBNK cells after treatment with Daratumumab (PBNK-CD38^−^) for 48 h, compared to the untreated population. (**c**) FACS phenotypic analysis comparing the PBNK cells treated and not treated with Daratumumab for 48 h. (**d**) Graph with the cytotoxicity of pretreated PBNK-CD38^−^ cells and original population with heterogeneous expression of CD38 against RPMI 8226 with Daratumumab. Combinatorial cytotoxicity was measured using the bioluminescence imaging assay at 1:1 effector-to-target ratio for 24 h. Data in (**b**) are means ± SEM of three expanded PBNK cell donors. Data in (**a**,**d**) are presented as means ± SD of each expanded PBNK cells in three technical replicates. *, *p* < 0.05, **, *p* < 0.01, ***, *p* < 0.001, based on Student’s *t*-test.

To properly evaluate the synergistic effect of combinatorial therapy in expanded PBNK cells with a different ratio of CD16 alleles (154/3G8), we explored the tumor cytotoxicity of PBNK cells bearing a high (0.46) and a low ratio (0.24) against the MM cell line RPMI 8226 after mixing with the approved mAb Daratumumab. Daratumumab is an IgG_1_ humanized mAb that recognizes overexpressed antigen CD38 in MM tumor cells, causing their death through antibody-dependent phagocytosis, complement-dependent cytotoxicity, blocking of CD38, and ADCC [41,42,43,44]. As expected, a significant increase in specific lysis of RPMI 8226 cells was observed in the combinatorial therapy with PBNK cells and Daratumumab, particularly in PBNK cells with a higher ratio (0.46) (Figure 4a).

We performed two parallel control experiments to prove ADCC specificity of Daratumumab. First, we cocultured PBNK cells and Daratumumab with the MM cell line U266, which lacks the expression of CD38. As shown in Figure 4a, no statistical differences were observed compared to the control without Daratumumab. In the second control, RPMI 8226 cells and PBNK cells were cocultured with isotype-matched human IgG_1_ antibody (Sigma). Compared to the control without mAbs, no statistical differences were observed (data not shown). These results demonstrate the convenience of selecting the appropriate FcγRIII allotype in PBNK cells in a combinatorial therapy with mAbs.

One main concern in the combinatorial therapy with PBNK cells and Daratumumab is the NK-cell depletion due to the surface expression of CD-38 in the NK cells itself. This phenomenon, named fratricide, results in NK self-destruction by ADCC [45]. To avoid this undesired event in the effector cells, we performed an in vitro pretreatment of the PBNK cells with Daratumumab. The expanded PBNK cells have a heterogeneous expression of CD38, with more than half of the population CD38 positive (~57%). Short (48 h) cultivation of the PBNK cells with Daratumumab caused the fratricide of the CD38^+^ PBNK cells, leaving only the population of PBNK cells negative for the expression of CD38 (PBNK CD38^−^).

The resulting PBNK CD38^−^ cells had a similar proliferation rate (Figure 4b) and phenotype (Figure 4c) as the original heterogeneous population for CD38.

Moreover, in order to assess the cytotoxicity of PBNK CD38^−^ cells in a combinatorial therapy with Daratumumab, we performed a cytotoxic assay against the RPMI 8226 tumor cell line and compared the results with the original PBNK cell population (not pretreated with Daratumumab). As predicted and shown in Figure 4d, Daratumumab’s combination with PBNK CD38^−^ cells induced higher specific cytotoxicity than the one observed with the original PBNK cells not pretreated and heterogenous for the expression of CD38.

Taken together, these data demonstrate how the efficacy of expanded PBNK cells against the MM cell line RPMI 8226 can be synergistically increased with mAb Daratumumab. To make the combinatorial therapy even more robust against MM cancer cells, we propose selecting a specific allotype for the receptor CD16 and a novel pretreatment of the PBNK cells with Daratumumab to avoid fratricide.

### 3.5. Efficacious Combinatorial Therapy with Expanded PBNK Cells and Monoclonal Antibody in Primary MM Cells and an MM Mouse Model

**Figure 5:**
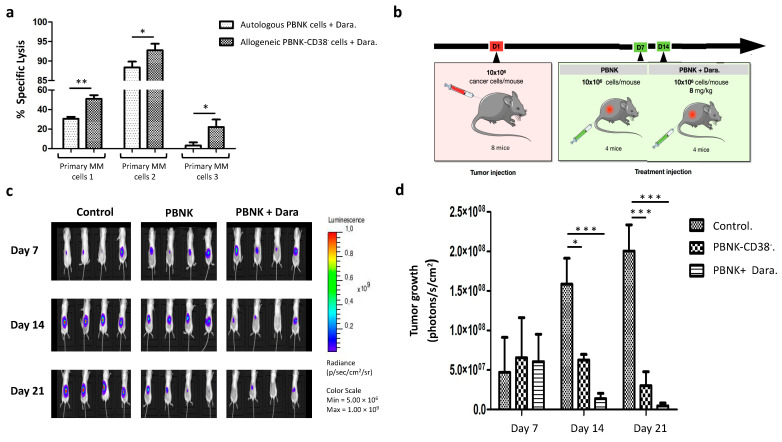
Combinatorial therapy in primary MM cells and in vivo mouse model of MM. (**a**) Graph with the specific lysis results in a combinatorial therapy with allogeneic PBNK-CD38^−^ and autologous PBNK cells against primary MM cells. Measured by flow cytometry at a 5:1 effector-to-target ratio for 4 h. (**b**) Schematic depiction of treatment regimen in the different groups. After tumor implantation on day 1, mice were divided into three groups: control group (*n* = 4), single-agent treatment group with PBNK-CD38^−^ cells (*n* = 4), and combo-treatment group with Daratumumab and PBNK-CD38^−^ cells (*n* = 4). (**c**) Representative bioluminescence images in each group at 7, 14, and 21 after RPMI 8226 cell inoculation. (**d**) Summary graph with quantification of tumor growth over time (*n* = 4 per group) by bioluminescence, demonstrating the higher inhibition of RPMI 8226 progression by the combinatorial therapy than control and single-agent groups. Data in (**a**) are presented as means ± SD from three technical replicates. Data in (**d**) are ± SEM. *, *p* < 0.05, **, *p* < 0.01, ***, *p* < 0.001, according to one-way ANOVA, repeated measures test, and Student’s *t*-test.

To assess the translational relevance of expanded PBNK-CD38^−^ cells (R > 0.3) for the treatment of patients with MM in combination with Daratumumab, we studied their cytotoxic capacity against primary MM cells from newly diagnosed patients. The cytotoxic effect was compared to PBNK cells from the same patient (autologous) combined with Daratumumab. As shown in Figure 5a, allogeneic PBNK-CD38^−^ cells (R > 0.3) combined with Daratumumab exerted cytotoxicity against the three primary MM samples. Compared to autologous PBNK cells, the specific lysis observed in allogeneic PBNK-CD38^−^ cells was significantly higher in all three cases.

To evaluate the therapeutic efficacy of expanded PBNK-CD38^−^ cells (R > 0.3) combined with Daratumumab in vivo, we applied the combinatorial therapy in mice bearing a subcutaneous MM model with the RPMI 8226 cell line previously labeled for the constitutive expression of luciferase and the fluorescent protein mCherry. We compared the combinatorial therapy with single-agent therapy to estimate the synergy of PBNK cells with Daratumumab. A schematic diagram of the treatment regimen in the three groups of mice is shown in Figure 5b; control group without treatment, single-agent treatment with PBNK-CD38^−^ cells, and combinatorial treatment group with Daratumumab and PBNK-CD38^−^ cells. The tumor progression of each group was tracked over time by bioluminescence (Figure 5c). Serial bioluminescence imaging showed that the combinatorial therapy with expanded PBNK-CD38^−^ cells (R > 0.3) and Daratumumab significantly reduced tumor growth by day 14, resulting in MM tumor volumes that were 43-fold smaller than in control mice by day 21 (Figure 5c,d). Average tumor volumes in single treatment with PBNK-CD38^−^ cells were 6.6 fold smaller than in the control group by day 21.

Accordingly, the in vivo combinatorial therapy with expanded PBNK-CD38^−^ cells (R > 0.3) and Daratumumab exerted a synergistic and robust anticancer effect against MM cancer cells, significantly higher than that observed with a single agent.

## 4. Discussion

Adoptive cell therapy or cellular immunotherapy has shown encouraging results in the treatment of different tumors [46,47,48]. Nevertheless, it is still a fledgling, with a long road ahead, not exempt from roadblocks, such as toxicity [3], tumor-homing [49], tumor escape [50], and limited by expenses and time needed to manufacture autologous cells [51]. Therefore, in this study, we explore the use of allogeneic “off the shelf” NK cells as a cell source of fully functional effector cells capable of circumventing the obstacles in adoptive cell therapy.

The NK cells can be found in umbilical cord blood [52] and peripheral blood [53], and they can also be derived from stem cell sources such as human pluripotent cells [54] and umbilical cord blood hematopoietic stem cells [55]. We decided to explore the peripheral blood-derived NK cells for two main reasons. First, compared to other cell sources, such as those derived from umbilical cord blood and hematopoietic stem cells, a high amount of NK cells can be obtained from one single donor, achieving clinically relevant NK cell numbers by simple methods of expansion. The second reason is that in contrast to NK cells derived from induced pluripotent stem cells [56], no adverse effects have been reported after administration [57,58].

Considering the potential risk of GvHD, we depleted non-NK cells from peripheral blood with an untouched NK cell isolation kit, also available in GMP grade. With this method, we yielded similar NK cell numbers from different donors with very high purity. We expanded and carefully phenotyped the isolated PBNK cells from three different donors to obtain clinically relevant numbers and ensure reproducibility for future clinical applications. To date, the methods for NK cell expansion can be divided into the ones that use feeder cells or do not. Though results are promising in expanding NK cells with feeder cells [59], they produce an extra layer of complexity in future scale-up applications and raise safety concerns when tumor cell lines are used as feeder cells. For these reasons, we decided to explore the expansion of PBNK cells in the GMP-grade feeder-free medium, optimized for NK cell culture (NK MACS Medium) with defined cytokines IL-2 and IL-15. Applying this expansion method, we obtained highly purified and clinically relevant NK cell numbers, with a median of 14,050 million PBNK cells from one donor after five weeks of expansion. Interestingly, the expansion median folds were higher compared to a previous study with a feeder-free medium with IL-2 and IL-5 after one week of expansion (median of 1-fold vs. median of 5.24-fold) [60]. The differences were even higher when compared to another previous study with feeder-free medium supplemented just with IL-15 after three weeks of cultivation (median of 23-fold vs. median of 196-fold) [61].

In contrast to other studies, we performed a longitudinal study of the PBNK cell’s tumor-killing properties in different tumor types and at different time points of expansion. We found that expanded PBNK cells have a multi-tumor target capacity and achieved maximum killing capacity in the fourth week of expansion. The observed increase in the killing capacity of our PBNK cells may be explained, at least in part, by increased expression of the activation receptor NKG2C. Besides, we observed that expanded PBNK cells presented a high expression of the activation receptor NKp44, which after engagement with the growth factor PDGF-DD produced by tumor cells, induced IFN-γ expression. On the other hand, we observed a slight decline in the killing capacity of PBNK cells towards different tumor cell lines in the sixth week of expansion, most likely due to prolonged exposure to IL-15 that was reported to entail NK cell exhaustion [29].

The tumor-homing properties of expanded PBNK cells are essential to guarantee the desired therapeutic effect. The molecular mechanism mediating NK cell homing to tumor cells is based on the chemotaxis of NK cells to the gradient of chemokines secreted by tumor cells and the tumor microenvironment [34]. We found that the PBNK cells express prime chemokine receptors, such as CXCR3, CXCR4, CCR5, and CXCR6, that match chemokines produced by the studied tumor cells. Unlike other investigations that studied this phenomenon in one or few tumor types, we proved the tumor-homing of expanded PBNK cells in a large number of solid and liquid tumor cell types in parallel. These results will pave the way for future investigations to improve tumor tropism of expanded PBNK cells towards specific tumor cell types.

To further increase the effectivity of expanded PBNK cells against incurable MM, we decided to explore their combination with mAbs as an alternative approach to genetic manipulations, as NK cells are extremely resilient to common transfection or infection methods [62]. With such an approach, we also ensured easy implementation in future clinical applications. MM accounts for 1% of all cancers and is the 2nd most common hematologic malignancy after lymphoma and leukemia [63]. MM is characterized by proliferation and accumulation of aPCs, increasing monoclonal immunoglobulin levels in serum or urine, and evidence of end-organ damage [64,65]. The typical clinical manifestations of MM and referred to as CRAB, are hypercalcemia67, renal failure [66], anemia [67], and osteolytic bone lesions [68,69].

The outcome of patients with MM has significantly improved over the past decade due to novel therapeutic agents such as proteasome inhibitors, immunomodulatory drugs, mAbs, and autologous stem cell transplantation [70]. Nevertheless, despite high treatment efficacy, most of the patients eventually relapse, with a short progression-free survival period [71,72,73]. In the particular case of the clinically approved mAb Daratumumab, the pharmacological effect is due to the direct induction of apoptosis, ADCC, antibody-dependent cellular phagocytosis, and complement-dependent cytotoxicity [41]. However, patients with MM often bear immune dysfunction with poor NK cell cytotoxic functions due to an increased expression of CD57^+^ (terminally differentiated marker), killing inhibitor receptors, and reduced expression of activating receptors [22]. Because the ADCC with Daratumumab is mainly mediated by NK cells, Daratumumab’s therapeutic effect in MM patients can be severely compromised.

Thus, we proposed combining Daratumumab and fully functional allogenic PBNK cells to overcome patients’ NK cell dysfunctionality and obtain a synergistic effect in combinatorial therapy. As we expected, a significant increase in killing capacity against MM cell lines was achieved with PBNK cells in combination with Daratumumab.

To increase the ADCC effect, PBNK cells were assessed for the expression of specific CD16 polymorphism with a stronger affinity to the mAb Daratumumab [39]. We found that expanded PBNK cells combined with Daratumumab and selected for specific CD16 polymorphism expression can exert a significantly higher immune response against MM cell lines. To further increase the synergistic effect, we invented a new and facile in vitro pretreatment method to circumvent fratricide in a combinatorial approach with Daratumumab and NK cells. Our straightforward method avoids complex, expensive, and time-consuming approaches such as the one presented by Meisam Naeimi Kararoudi et al., which requires genetic manipulation to delete CD38 expression in NK cells [74]. With our uncomplicated approach and in only two days, we obtained a homogenous CD38^−^ NK cell population capable of bypassing fratricide and significantly boosting the NK cells’ effector activity against MM cells lines in combination with Daratumumab. Importantly, the translational potential of the therapeutic expanded allogeneic PBNK-CD38^−^ cells in combination with Daratumumab was also proved against primary MM cells.

## 5. Conclusions

Our in vitro and in vivo findings presented in this study demonstrate the feasibility of using allogeneic PBNK cells as a cell source for treating different types of cancer. We highlight their potential in a combinatorial therapy to treat MM with mAbs after donor CD16 polymorphism selection and novel in vitro pretreatment to avoid fratricide. Our results fully support and justify continued development of allogeneic PBNK cells for translation into clinical settings in combination with mAbs to overcome patients’ NK cell dysfunctionality and to obtain a synergistic effect in combinatorial therapy.

## Data Availability

The data presented in this study are available from the corresponding author upon request.
